# Improved outcome of haploidentical transplantation in severe aplastic anemia using reduced-intensity fludarabine-based conditioning

**DOI:** 10.18632/oncotarget.19745

**Published:** 2017-07-31

**Authors:** Wu Yamei, Luo Rongmu, Cao Yongbin, Si Yingjian, Li Xiaohong, Zhang Xiaomei, Yan Pei, Du Zhenlan, Wang Haitao, Wang Jing, Wang Bojing, Wu Xiaoxiong, Da Wanming

**Affiliations:** ^1^ Department of Hematology, The First Affiliated Hospital, Chinese PLA General Hospital, Beijing 100048, China; ^2^ Department of Hematology, Affiliated BaYi Children's Hospital, PLA Army General Hospital, Beijing 100700, China; ^3^ Department of Hematology, Chinese PLA General Hospital, Beijing 100853, China

**Keywords:** haploidentical, hematopoietic stem cell transplantation, severe aplastic anemia, graft-versus-host disease, graft failure

## Abstract

Significant improvements in hematopoietic stem cell transplantation (HSCT) with haploidentical family donors (HFD) have confirmed its therapeutic role in severe aplastic anemia (SAA) and led to the evolution of treatment algorithms. However, the optimal conditioning regimen for HFD-HSCT remains undefined, especially the dosage of cyclophosphamide (Cy).

A total of 77 patients with SAA from two research centers, who received HFD-HSCT with reduced-intensity fludarabine + cyclophosphamide + thymoglobulin ± busulfan conditioning regimen plus third-party cells infusion were included in this study, of which 67 pairs had 4-5 loci mismatched. We were particularly interested in whether the dosage of Cy significantly impacted graft failure (GF) and overall survival (OS).

All patients showed sustained hematopoietic engraftment without any increase in severe aGVHD and transplantation-related mortality (TRM). The incidences of grade II-IV aGVHD, grade III-IV aGVHD and extensive cGVHD were 18%, 10% and 7%, respectively. The probabilities of 1-year and 5-year OS were 93.1% and 87.9%, respectively. Furthermore, patient age <15 years, MNC cells >8×10^8^/kg and donor age <45 years were associated with better survival (*P*=0.043, *P*=0.023, and *P*=0.037, respectively) and engraftment (*P*=0.019, *P*=0.008, and *P*=0.001, respectively).

Our findings indicated that SAA patients lack MSD benefited the most if HFD-HSCT was performed with reduced-intensity fludarabine-based conditioning regimen. Improved outcomes with HFD-HSCT may lead to a salvaged therapy and an expanded direct role for SAA in the future.

## INTRODUCTION

Severe aplastic anemia (SAA) is a rare multi-lineage bone marrow failure and life-threatening disorder, with a high annual incidence of nearly 8% of all malignant and nonmalignant hematological disorders in China [1, 2]. Hematopoietic stem cell transplantation (HSCT) from a human leukocyte antigen (HLA)-matched sibling donor (MSD) has become the first-line and gold-standard initial treatment option for younger patients, with long-term survival up to 95% [3–5]. When an MSD is unavailable, intensive immunosuppressive therapy (IST) with horse anti-thymocyte globulin (ATG) and cyclosporine (CSP) is indicated [6]. However, IST has a high failure rate, due to lack of response, relapse and clonal evolution, which was recently demonstrated in two large pediatric series from Europe and Japan that showed a much lower failure-free survival (FFS) following IST than MSD HSCT (37-56% post-IST versus 83-87% post-MSD HSCT) [7, 8]. Treating patients with SAA who lack suitable MSD or matched unrelated donor (MUD) is challenging, particularly those with heavy transfusion and refractory or relapse of IST [9]. Improvements in donor selection, conditioning protocols and supportive care along with advances in understanding disease biology have contributed to increasing success rates of HSCT with alternative donors [10, 11].

During the past two decades, alternative donor transplantations using haploidentical family donors (HFD) were suggested to be potential treatments for SAA patients requiring frequent transfusions and repeated IST [12–16]. However, this procedure is limited by a high risk of transplantation-related mortality (TRM), severe high rates of graft rejection (GR), graft-versus-host disease (GVHD) and low overall survival (OS). Recent advances in effective *ex vivo* depletion of T-cells or unmanipulated *in vivo* regulation of T-cells, better supportive care, and evolving conditioning regimens have significantly improved the outcome of HFD-HSCT [12–17]. The T-cell antibody, ATG, is critical for *in vivo* T-cell depletion (TCD). The conditioning regimen of fludarabine (Flu), cyclophosphamide (Cy) and ATG combination (FC/ATG) has been used in patients with SAA not only in MSD transplants but also in alternative donor HSCT [9–14]. However, the optimal conditioning regimen for HFD-HSCT remains undefined, especially the dosage of Cy (range: 50–200 mg/kg), which may be associated with TRM, GF and OS. Furthermore, majority of the studies have included either ≤ 30 cases or single-group with short-term follow-up, making the results difficult to interpret. Our center had used infusion of umbilical cord tissue-derived MSCs (UC-MSCs) as pilot salvage therapy for 21 patients undergoing HFD-HSCT with 2-year OS up to 75% [13]. Based on our prior successful experiences, we pioneered the reduced-intensity fludarabine-based conditioning with third-party cells infusion for treatment of SAA, especially for patients with heavy transfusion and long-term IST, with the following two features: first, reduction of the dosage of Cy decreased or alleviated the toxic side-effects, for myeloablative and effective engraftment. Second, third-party donor-derived cells were infused to prevent GVHD and improve the hemato-immunological reconstitution. In the current study, we investigated the long-term feasibility and clinical value of HFD-HSCT with reduced-intensity fludarabine-based regimen plus third-party cells infusion to treat SAA in 77 patients from two research centers.

## RESULTS

### Patient and donor characteristics

Characteristics of patients with HFD-HSCT and donors prior to HSCT are presented in Table 1. Median age of 77 patients and donors were 11 years (range, 1-46 years) and 34 years (range, 11-57 years), respectively. All patients received IST prior to referral to both centers for HFD-HSCT (ATG included; n=15 and ATG excluded; n=62). Notably, all donors were HLA-mismatched related (2 HLA loci mismatched; n=5; 3 HLA loci mismatched; n=5; 4 HLA loci mismatched; n=23; and 5 HLA loci mismatched; n=46). Of the total HFD-HSCT, 40 were performed with regimen A and the remaining 37 with regimen B. The median MNC dose infused was 10.10×10^8^/kg (range, 6.30-33.63×10^8^/kg, Table 1), while the median CD34 stem cell dose was 4.77×10^6^/kg (range, 1.01-17.72×10^6^/kg, Table 1A).

Table 1

**Table:** A

Variable	Data
**Median age at diagnosis [yr](range)**	8(1-45)
**Disease, n(%)**	
SAA or VSAA	72(93)
SAA&PNH	5(7)
**IST prior to referral for HSCT, n(%)**	
ATG included	15(19)
ATG excluded	62(81)
**Transfusion RBC before HSCT, U(%)**	
≥25U	29(38)
<25U	48(62)
**Median age at transplant [yr](range)**	11(1-46)
≤15yr, n(%)	52(67)
>15yr, n(%)	25(33)
**Median disease duration before HSCT[m](range)**	7(2-182)
**Sex(female/male)**	38/39
**Source of stem cells, n(%)**	
BM+PB	77(100)
**Regimen, n(%)**	
A(Flu+Cy+ATG+Bu)	42(54)
B(Flu+Cy+ATG)	35(46)
**Median MNCs, ×10^8^/kg (range)**	10.10(6.30-33.63)
**Median CD34+, ×10^6^/kg (range)**	4.77(1.01-17.72)
**Engraftment, n(%)**	
Primary engraftment	74(96)
Secondary engraftment	3(4)
**Median time of ANC ≥0.5 × 10^9^/L [d](range)**	12(8-21)
**Median time to PLT ≥20 × 10^9^/L [d](range)**	14(9-30)
**aGVHD, n(%)**	
Grades II-IV	20(26)
Grade III-IV	8(10)
**cGVHD, n(%)**	
Limited	14(18)
Extensive	5(7)
**Median follow-up time [m](range)**	20(1-66)

**Table:** B

Variable	Data
**Median age [yr](range)**	34(11-57)
**D-R sex match, n(%)**	
Female to female	21(28)
Female to male	18(23)
Male to female	11(14)
Male to male	27(35)
**D-R major blood group match, n(%)**	
Match	43(56)
Mismatch	34(44)
**D-R relationship, n(%)**	
Parent-child	66(86)
Child to parent	2(3)
Siblings	9(11)
**D-R HLA-loci mismatched, n(%)**	
2 HLA loci	4(5)
3 HLA loci	4(5)
4 HLA loci	23(30)
5 HLA loci	46(60)

D-R, donor-recipient.

### Engraftment, chimerism and graft failure

Data for all patients who survived at least 30 days after HSCT and were evaluable for engraftment are summarized in Table 1A. The median time to neutrophil engraftment was 12 days (range, 8-21 days, Table 1). Patients in both groups achieved platelet engraftment in a median of 14 days (range, 9-30 days, Table 1). Two patients in regimen A and one patient in regimen B with temporary engraftment after primary graft rejection in regimen A group achieved secondary engraftment and underwent HFD-HSCT with another family donor (Table 2A). Secondary thrombocytopenia occurred in one patient in group A at day +92. The patient received additional donor lymphocyte infusion (DLI), and was alive and well. All patients achieved full donor chimerism by day +30 after HFD-HSCT.

Table 2

**Table:** A

Patient number	1	31	34
**Age**	16	46	20
**Diagnosis-to-transplant interval [m]**	50	7	100
**Donor-recipient HLA mismatched**	3	4	5
**Infused MNC cells(8×10^8^/kg)**	7.5	7.1	8.7
**Rejection type**	Rej	Rej	Rej
**Comment**	2 HFD-HSCT	2 HFD-HSCT	2 HFD-HSCT
**Autologous recovery**	No	No	No
**Alive(A)/Death(D), reason**	D/CMV-IPn	**A**	**A**
**Follow-up [m]**	5	18	9
**Transfusion dependent**	-	No	No

**Table:** B

Effects	Event(GF)/total	*p* value
**Patient age at transplant**		
<15 yr	0/49	**0.019**
≥15 yr	3/28	
**Diagnosis-to-transplant interval**		
≤6 months	0/38	0.081
>6 months	3/39	
**IST prior to referral for HSCT**		
ATG included	0/15	0.385
ATG excluded	3/62	
**Transfusion RBC before HSCT**		
≥25U	2/29	0.290
<25U	1/48	
**Infused MNC cells**		
≤8×10^8^/kg	2/11	**0.008**
>8×10^8^/kg	1/66	
**Infused CD34+ cells**		**0.001**
≤3×10^6^/kg	3/14	
>3×10^6^/kg	0/63	
**Conditioning regimen**		0.667
A(Flu+Cy+ATG+Bu)	2/42	
B(Flu+Cy+ATG)	1/35	
**Donor age**		**0.001**
<45 yr	0/65	
≥45 yr	3/12	
**D-R sex match**		0.290
Match	1/48	
Mismatch	2/29	
**D-R major blood group match**		0.700
Match	2/43	
Mismatch	1/34	
**D-R HLA mismatched**		0.341
2-4 HLA loci	2/31	
5 HLA loci	1/46	

(A) Clinical data and outcome of three patients with GR or GF. (B) Comparison of effects of different variables on GF of patients after transplantation.

GF, graft failure; GR, graft rejection.

D-R, donor-recipient.

Comparing the effects of different factors on SAA, GF is depicted in Table 2B. GF was significantly affected by patient age at transplant (*P* =0.019), donor age (*P* =0.001), the number of infused MNC cells (*P* =0.008) and the number of infused CD34+ cells (*P* =0.001). Moreover, time-to-transplant interval and RBC transfusion before HSCT may be associated with the incidence of GF potency, although not statistically significant (*P* = 0.081 and *P* = 0.290, respectively). However, there was no obviously decreasing tendency toward GF potency in patients with regimen A plus BU (*P* = 0.667).

### GVHD

Grade II-IV acute graft-versus-host disease (aGVHD) occurred in 20 patients (26%), of which n=11 (26%) were in group A, and n=9 (26%) in group B, and grade III-IV aGVHD occurred in eight patients (10%), of which n=5 (12%) were in group A and n=3 (9%) in group B. Of these, four patients died, and three progressed to extensive chronic graft-versus-host disease (cGVHD). Collectively, four patients from group A and one patient from group B developed extensive cGVHD, of which three died. Results of Chi-square statistical analysis with grade III-IV aGVHD and extensive cGVHD as the events of interest are presented in Table 4. Donor-recipient HLA mismatched 5 loci was associated with increased risk of grade III-IV aGVHD occurrence (*P*= 0.014, Table 4). For extensive cGVHD, donor age ≥45yrs and transfusion RBC ≥25U before HSCT were both significant predictors (*P* = 0.003 and *P* = 0.034, Table 4).

**Table 4:** Comparison of effects of different variables on acute and chronic GVHD occurrence after transplantation

Variable	aGVHD (grades III-IV)		cGVHD (extensive)	
**Event/total**	***P*** **value**	**Event/total**	***P*** **value**
**Patient age at transplant**		0.944		0.247
<15 yr	5/49		2/48	
≥15 yr	3/28		3/27	
**Diagnosis-to-transplant interval**		0.969		0.291
≤6 months	4/38		1/38	
>6 months	4/39		3/37	
**Patient sex**		0.146		0.174
Female	2/38		1/37	
Male	6/39		4/38	
**IST prior to referral for HSCT**		0.142		0.738
ATG included	0/15		1/14	
ATG excluded	8/62		3/61	
**Transfusion RBC before HSCT**		0.126		**0.034**
≥25U	5/29		4/27	
<25U	3/48		1/48	
**Conditioning regimen**		0.633		0.239
A(Flu+Cy+ATG+Bu)	5/42		4/41	
B(Flu+Cy+ATG)	3/35		1/34	
**Time to neutrophil engraftment**		0.907		0.174
≥12 days	4/40		4/38	
<12 days	4/37		1/37	
**Time to platelet engraftment**		0.746		0.456
≥14 days	5/44		2/42	
<14 days	3/33		3/33	
**Donor age**		0.438		**0.003**
<45yr	6/65		2/64	
≥45 yr	2/12		3/11	
**D-R sex match**		0.992		0.949
Match	5/48		3/46	
Mismatch	3/29		2/29	
**D-R major blood group match**		0.689		0.456
Match	5/43		2/42	
Mismatch	3/34		3/33	
**D-R HLA mismatched**		**0.014**		0.316
2-4 HLA loci	0/31		1/31	
5 HLA loci	8/46		4/44	

D-R, donor-recipient.

### Transplant-related complications

As seen in Table 3, toxicity of the reduced-intensity conditioning regimen was minimal. Among the 77 patients, only six (8%) experienced grade III TRT, while none developed grade IV TRT. One patient had grade III TRT in the liver and another patient had grade III TRT in the kidney, which may be associated with oral CSP for more than 15 years. One 5-year-old patient experienced cyclosporine-associated posterior reversible encephalopathy syndrome (CSP-associated PRES) when voriconazole was administered for invasive fungal disease. In addition, the incidences of hemorrhagic cystitis (HC) and oral ulcers obviously decreased, due to the reduced doses of cyclophosphamide.

**Table 3:** Transplantation-related toxicity following HFD-HSCT

Variable	Grade I/II, n(%)	Grade III/IV, n(%)
**Mucosa**	8	2
**Bladder**	1	0
**Kidneys**	2	1
**Liver**	2	1
**CNS**	1	1
**Heart**	1	0
**GI toxicity**	3	1
**total**	18(24%)	6(8%)

CNS, central nervous system; GI, gastrointestinal.

In terms of early virus reactivation (≤100d), viremia occurred in 42 patients (55%) and only one patient with secondary HFD-HSCT progressed to CMV-associated interstitial pneumonitis (IPn) after pre-emptive treatment. Epstein-Barr virus (EBV) viremia occurred in 18 patients (23%), of which one patient developed EBV-associated post-transplant lymphoproliferative disorder (PTLD), and was cured with DLI and rituximab. Out of 74 patients followed up for >100d, three developed CMV viremia, one EBV viremia and one PTLD.

Seven patients died after HSCT with a median time of 5 months (range 1-25 months). Overall, seven patients died of severe infections at 1 month, 4 months, 5 months and 17 months (one with septicemia, one with CMV-associated IPn and two with IFD), 3 months for severe aGVHD, 7 months for thrombotic microangiopathy (TMA) and 25 months for bronchiolitis obliterans organizing pneumonitis (BO) after HSCT.

### Survival

**Figure 1:**
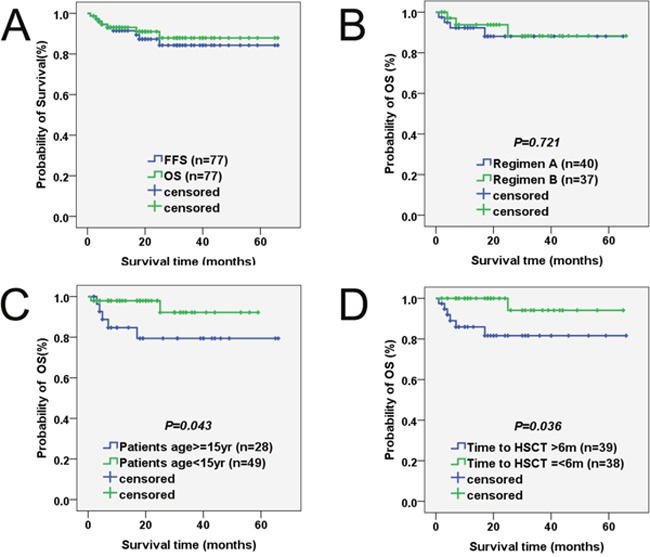
Probability of survival in patients with severe aplastic anemia undergoing HSCT with HFDs **(A)** OS and FFS. The probabilities of 1-year OS (green group) and 1-year FFS (blue group) were 93.1% and 91.5%, respectively. **(B)** Survival according to conditioning regimen. The 1-year OS rates between regimen A (blue group) and regimen B (green group) did not significantly differ (92.3% versus 93.8%, *p* =0.721). **(C)** Survival according to patient age at transplant. The 1-year OS rates between patients ≥15 years old (blue group) and patients <15 years old (green group) was significantly different (84.7% versus 97.9%, *P* =0.043). **(D)** Survival according to diagnosis-to-transplant interval. Statistical analysis revealed that the 1-year OS rates was obviously different between intervals >6 months (blue group) and intervals ≤6 months (green group) (85.9% versus 94.1%, *P*=0.036).

**Figure 2:**
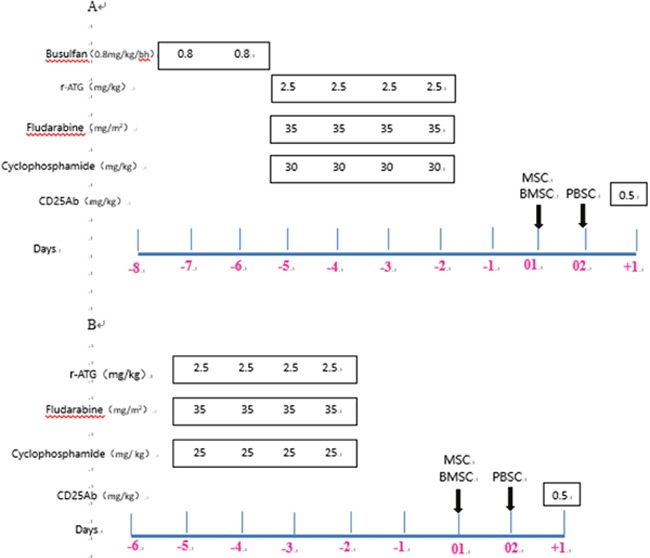
Transplantation protocol for severe aplastic anemia r-ATG, rabbit anti-thymocyte globulin, Sanofi; r-ATG-F, rabbit anti-thymocyte globulin, Fresenius. **(A)** Conditioning regimen A for patients with SAA and PNH, or heavy transfusion (RBC ≥25U), or failed r-ATG therapy. **(B)** Conditioning regimen B for other patients with SAA or VSAA.

During the follow-up for 1–66 months, 70 of the 77 (91%) patients were alive and well. As shown in Figure 2, the probabilities of 1-year, 2-year and 5-year OS were 93.1%, 91.0% and 87.9% in the 77 patients, respectively. Moreover, the probabilities of 1-year, 2-year and 5-year FFS were 91.5%, 87.3% and 84.3%, respectively. In addition, the 1-year OS rates did not significantly differ between groups A and B (92.3% versus 93.8%, *P* =0.721; Figure 1).

Comparing the effects of different factors on SAA, OS is depicted in Table 5 and Figure 1. Chi-square statistical analysis revealed that intervals >6 months was a significant predictor of death (*P* =0.036). Age of patients and donors at transplant was significantly associated with patient outcomes after transplantation. Better outcomes were seen in patients <15 years old (*P* = 0.043) and donors <45 years old (*P* = 0.037) at transplant than those who were older. Moreover, patient outcomes were also significantly affected by the number of infused MNC cells (*P* =0.023). Similarly, a significantly increasing tendency toward worse OS was noted in patients who developed grade III-IV aGVHD or extensive cGVHD (*P*=0.001 and *P*=0.001, respectively).

**Table 5:** Comparison of effects of different variables on OS of patients after transplantation

Effects	Event(death)/total	*p* value
**Patient age at transplant**		
<15 yr	2/49	**0.043**
≥15 yr	5/28	
**Diagnosis-to-transplant interval**		
≤6 months	1/38	**0.036**
>6 months	6/39	
**Patient sex**		0.249
Female	2/38	
Male	5/39	
**IST prior to referral for HSCT**		
ATG included	2/15	0.524
ATG excluded	5/62	
**Transfusion RBC before HSCT**		
≥25U	4/29	0.265
<25U	3/48	
**Infused MNC cells**		
≤8×10^8^/kg	3/11	**0.023**
>8×10^8^/kg	4/66	
**Infused CD34+ cells**		0.076
≤3×10^6^/kg	3/14	
>3×10^6^/kg	4/63	
**Conditioning regimen**		0.721
A(Flu+Cy+ATG+Bu)	4/42	
B(Flu+Cy+ATG)	3/35	
**Time to neutrophil engraftment**		
≥12 days	4/40	0.773
<12 days	3/37	
**Time to platelet engraftment**		0.423
≥14 days	5/44	
<14 days	2/33	
**aGVHD**		**0.001**
No/Grades I-II	3/69	
Grades III-IV	4/8	
**cGVHD**		**0.001**
No/Limited	4/72	
Extensive	3/5	
**Donor age**		**0.037**
<45 yr	4/65	
≥45 yr	3/12	
**D-R sex match**		0.684
Match	4/48	
Mismatch	3/29	
**D-R major blood group match**		0.468
Match	3/43	
Mismatch	4/34	
**D-R HLA mismatched**		0.508
2-4 HLA loci	2/31	
5 HLA loci	5/46	

D-R, donor-recipient.

## DISCUSSION

Despite promising evidence for HFD-HSCT in acquired SAA in the last decade, several obstacles remain, such as standardized conditioning regimen, methods to regulate the donor T-cells and decrease GVHD, stem cell source, donor selection, and management of GF or poor graft function, which need to be resolved for widespread use [23, 24]. In this study, patients with SAA grafted from HFD, who were administered a reduced-intensity fludarabine-based conditioning regimen, with third party cells infusion, showed an encouraging 2-year survival of 91.0%, without decrease in the engraftment potency and increase in incidence of GVHD. The new data in the study has been substantially improved to our publication of *Stem Cell Research*, and at the same time, which could achieve comparable outcomes with HLA-identical sibling transplantation our center (2-year survival of 93.0%, unpublished data). Furthermore, there were six major risk factors of OS, albeit with no significant repetition in multivariate analysis and this held true for other significant associations with OS and FFS. Indeed, 70 of the 77 (91%) patients with SAA who received HFD-HSCT had normal full blood counts and the majority had full donor chimerism, indicating complete restoration of normal bone marrow function.

Recently, fludarabine-based conditioning regimens for SAA patients receiving alternative donor HSCT have shown promising results, but the role and dose of Cy in the combination regimens have not been well established or fully defined [24–28]. The EBMT has shown that FC/ATG may be preferable to the conventional high dose Cy (200 mg/kg) for stable engraftment in SAA patients >30 years receiving an HLA-identical graft [25]. However, the EBMT has also shown that FC/ATG could use low dose Cy (1200 mg/m^2^, which is approximately equal to 40-50 mg/kg) for obviously decreasing TRM in very young patients with SAA receiving MUD-HSCT, of which GF was reported in 16-18% of the total sample [24, 26]. Tolar et al. proposed that a Cy dose combined with low-dose TBI should range between 50 and 100 mg/kg to prevent excessive organ toxicity and GF in MUD-HSCT [28]. Im et al. described 12 patients who underwent T-cell-depleted (TCD) HFD-HSCT after conditioning with Flu+Cy (120 mg/kg) + ATG ± TBI, where the incidence of GF decreased to 4.8% [29]. Xu *et al*. recently reported 101 patients from multiple centers with 4% incidence of GF and 10% rate of grade III/IV TRT, who underwent HFD-HSCT after Cy (200 mg/kg) + ATG protocol with additional BU [30]. In accordance with these findings and our experience, this study showed that Cy 100 mg/kg may be the most optimal dose in combination with Flu + ATG ± BU for HFD-HSCT for SAA with minimal GF potency (4%) and grade III/IV toxicity (8%). Moreover, primary GF caused by immunological resistance to the grafts or inadequate number of progenitors are more common in nonmalignant diseases including SAA, especially heavily transfused cases, than in acute leukemia [24, 31, 32]. For almost 50% of patients with heavy transfusion in this study, UC-MSCs and high-dose stem cell numbers were both administered to overcome GF and GR. Previous reports have also shown that MSCs could improve engraftment in poor engraftment or graft failure after HSCT [12, 20], as well as in patients who previously experienced graft rejection [11, 32]. In the current study, 74 of 77 patients (96%) achieved a rapid primary hematopoietic recovery and total full donor chimerism. In addition, the three cases with temporary engraftment after primary GR achieved secondary engraftment underwent HFD-HSCT with another family donor, of which two patients are still alive and well.

HSCT with HFD has been used as a salvage treatment for SAA patients who failed IST, with some encouraging outcome for reducing fatal GVHD in recent studies [12–16]. Numerous approaches have been previously attempted to reduce the incidence of GVHD after HSCT, such as depletion of CD3 cells (or selection of CD34 cells), additional infusion of MSC, intensive prophylactic program for GVHD, and reduced-intensity conditioning regimen. In our study, HFD-HSCT showed similar results to MSD HSCT [2, 8, 23, 33]. Taking MSD-HSCT and SAA into consideration, grade III-IV aGVHD, cGVHD and extensive cGVHD rates ranged from 10-20%, 20-44% and 7-20%, respectively [2, 34–36]. Based on this study, grade III-IV aGVHD, cGVHD and extensive cGVHD rates were 10%, 25% and 7%, respectively, and comparable to above-cited articles. Grade III-IV aGVHD and extensive cGVHD are lethal forms of GVHD. Grade I-II aGVHD does not frequently cause fatalities and the limited cGVHD effects eventually diminish, so the patients do not face serious problems, and quality of life post-transplantation is not altered. After reduced-intensity conditioning regimen, the GVHD prophylaxis consisted of CSP, MMF, ATG, CD25Ab and UC-MSCs. Meanwhile, G-CSF-primed bone marrow to the peripheral blood grafts were used, which might contribute to the low incidence of severe GVHD [37]. In our retrospective study, donor-recipient HLA mismatched 5 loci were at greater risk of grade III-IV aGVHD, while donor age ≥45 years and transfusion RBC ≥25U before HSCT were at greater risk of extensive cGVHD. Similar to OS, GVHD occurrence did not significantly correlate with previous IST treatment [2, 38].

The previous barriers to HFD-HSCT, delayed immune reconstitution, severe subsequent infections and TRM are considerably reduced with better supportive care and optimally modified conditioning regimens. The advent of high resolution tissue typing and novel conditioning regimens has significantly reduced the likelihood of these complications. Furthermore, our results suggested that reduced-intensity conditioning regimen might be beneficial for patients with a poor performance score. An additional potential barrier is the time lag between diagnosis and an MSD or MUD HSCT, due to difficulties in finding suitable donors and the time needed to arrange for a donation; which in turn could lead to continuous transfusion, a delay in neutrophil recovery and opportunistic infections [17, 39]. HFD transplantation could be immediately performed without racial or ethnic restrictions because virtually all patients who need HSCT can find a family donor. Furthermore, access to HFD is easier than MUD for cellular therapy to treat infections such as additional DLI for PTLD.

In summary, we found that all major endpoints of allogeneic HSCT for SAA, including engraftment, GVHD, life-threatening transplant-related complications, and OS were acceptable. Given the low TRM, stable engraftment and acceptable occurrence of GVHD, reduced-intensity fludarabine-based conditioning with third party cells infusion is considered an established option for patients with SAA by HSCT but lacking suitable MRD or MUD. The finding can potentially change our outlook toward HFD-HSCT for SAA patients, and should encourage further exchange of ideas and experience among transplant specialists. In addition, these findings must be validated by large sample-size, randomized, multi-center and prospective clinical trials.

## PATIENTS AND METHODS

### Patients and study design

This study enrolled 77 consecutive patients with SAA aged 1-46 years (median 9 years), referred to The First Affiliated Hospital of Chinese PLA General Hospital and Affiliated BaYi Children's Hospital, PLA Army General Hospital, who were treated with HFD-HSCT between January 2011 and June 2016. Patients or their legal guardians provided written informed consent for inclusion in the study. The clinical protocol and consent forms were approved by the institutional review board for human investigation at the two research institutions. All procedures described in this study were part of standard care at the two research institutions at the time of treatment.

The patients met the following criteria: (i) diagnosis of SAA, very SAA or SAA and paroxysmal nocturnal hemoglobinuria (PNH) according to the International Aplastic Anemia Study Group [18]; congenital forms of aplastic anemia were excluded; (ii) lack of human leucocyte antigen-MRD or MUD; (iii) no response to previous IST; (iv) absence of any severe pulmonary, cardiac, liver, or renal diseases or active infection; (v) adequate performance status [Eastern Cooperative Oncology Group (ECOG) score 0–2].

### Donors and HLA disparity

Donor-recipient HLA compatibility was pre-determined by high-resolution DNA techniques for detecting 10 of 10 alleles, including HLA-A, HLA-B loci, HLA-DRB1, HLA-DQB1 and HLA-C. Donors were ranked based on HLA match, age (younger preferred), gender (same preferred), adaptive binary optimization (ABO) compatibility, and health status (healthy preferred).

### Conditioning regimens

The reduced-intensity fludarabine-based conditioning included one of two regimens: (A) Patients had SAA and PNH, or heavy transfusion (RBC ≥25U), or failed rabbit ATG (r-ATG, Thymoglobuline, Sanofi, Genzyme Polyclonols S.A.S.) therapy, and received 0.8 mg/kg/6h busulfan (days -7 to -6), 35 mg/m^2^/day fludarabine (days -5 to -2), 25 mg/kg/day cyclophosphamide (days -5 to -2) and 2.5 mg/kg/day r-ATG (days -5 to -2). (B) Other patients with SAA or VSAA received 35 mg/m^2^/day fludarabine (days -5 to -2), 25 mg/kg/day cyclophosphamide (days -5 to -2), and 2.5 mg/kg/day r-ATG (days -5 to -2). A graphic representation of the conditioning regimens is shown in Figure 2.

### Allogeneic HSC infusion

Donor bone marrow (BM) and peripheral blood (PB) cells were collected using standard mobilization protocols. Granulocyte colony-stimulating factor (G-CSF, 5μg/kg/d for 5 days; Kirin Brewery, Tokyo, Japan) was used to mobilize BM stem cells and PB cells (G-BMPB). BM cells were harvested to achieve a target mononuclear cell count (MNC) of 2-4 × 10^8^/kg of recipient weight. The target MNC from PB was 4-6× 10^8^/kg of recipient weight. BM cells were intravenously infused through a central venous catheter on day 01, and thawed PB cells were infused on day 02.

### Third-party donor-derived cells infusion

UC-MSC were used as the third-party donor-derived cells for group A and group B cohorts, respectively. UC-MSC were purchased from the National Engineering Research Center of Cell Products, State Key Laboratory of Experimental Hematology. Immunophenotyping of UC-MSC was described by Lu et al [19, 20]. The planned UC-MSC dosage was 5.0 × 10^5^ cells/kg of recipient weight. BM infusions were performed 4 hours after the completion of UC-MSC infusion [13].

### Prophylaxis and treatment of GVHD

GVHD prophylaxis consisted of intravenous CSP 3 mg/kg/day in divided doses beginning on the day before transplantation (day -5) and the target concentration was adjusted to 200-250 ng/ml. Patients were advanced to oral CSP, as tolerated. In the absence of GVHD, the target concentration of CSP was administered for up to 1 year post-HSCT, then tapered and discontinued over the following 3-4 months. The oral MMF dose was 20 mg/kg/day from day −3 and was tapered off after 100 days if no aGVHD was observed. CD25Ab (Basiliximab, Novartis Pharma Stein AG, Switzerland) was also intravenously administered at a dose of 0.5 mg/kg/day only on day 1 after HSCT. Short-term methotrexate (MTX) was not given for GVHD prophylaxis. Acute and chronic GVHD were treated according to institutional practices.

### Engraftment, toxicity grading, and GVHD grading

Neutrophil engraftment was defined as the first of three consecutive days in which the neutrophil counts (ANC) exceeded 0.50 × 10^9^/L, and platelet engraftment was defined as the first of five consecutive days in which the platelet count exceeded 20 × 10^9^/L without transfusion. GF was classified as follows: (1) primary non-engraftment (failure to reach ANC of 0.5×10^9^/L after transplant); (2) rejection (decrease in blood ANC to <0.5×10^9^/L, after achieving ANC of 0.5×10^9^/L); (3) late graft failure (decrease in blood ANC after day 100 to <1.0×10^9^/L and platelets to <30×10^9^/L).

The transplantation-related toxicity (TRT) was graded using the National Cancer Institute Common Toxicity Criteria for Adverse Events version 4.0. Time of onset of TRT was defined as toxic effects occurring within 40 days after HSCT. Organ damage due to GVHD or infections were excluded. GVHD was defined according to established criteria [21]. OS was defined as the time from transplantation to death from any cause or the last follow-up. FFS was defined as survival with a response to therapy. Death, GF and relapse were considered as treatment failure. TRM was defined as death without disease progression.

### Supportive care and infection prevention

Antibiotics were empirically administered for fever and neutropenia according to institutional guidelines. G-CSF was routinely administered. Supportive care was provided according to individual institutional practices, and included cytomegalovirus (CMV) infection prophylaxis with ganciclovir and *Pneumocystis carinii* pneumonia prophylaxis with trimethoprim-sulfamethoxazole [22]. These treatments were administered after ANC exceeded 0.50×10^9^/L and continued until immunosuppressive therapy was discontinued. Antifungal prophylaxis included fluconazole, itraconazole, or liposomal amphotericin.

### Chimerism analyses

Chimerism was typically evaluated in recipient BM cells on days +30, +100, +180, and +365 after HSCT using cytogenetic G-banding or fluorescence *in situ* hybridization. Sex-matched donor-recipient chimerism was assessed using PCR-based analyses of polymorphic minisatellite or microsatellite regions. HLA typing was performed for patients with HLA-haploidentical donors.

### Statistical analyses

SPSS 21.0 statistical software was used for all statistical analyses. Patient, disease, and HSCT-related variables were compared between patients with regimens A and B using chi-square statistics for categorical variables and the Kruskal-Wallis test for continuous variables. Survival data were analyzed using the log-rank test, and survival curves were plotted using the Kaplan-Meier method. The incidences of aGVHD and cGVHD were evaluated using the Kaplan-Meier estimate of disease-free survival rates. Differences were considered significant at *P*< 0.05.
